# Effects of Wildfire on Soil CO_2_ Emission and Bacterial Community in Plantations

**DOI:** 10.3390/microorganisms12081666

**Published:** 2024-08-13

**Authors:** Yu Yang, Xuehui Liu, Shilin Huang, Jinchen Jia, Chuangye Wang, Lening Hu, Ke Li, Hua Deng

**Affiliations:** 1 Guangxi Key Laboratory of Environmental Processes and Remediation in Ecologically Fragile Regions, Guangxi Normal University, Guilin 541004, China; yangyu@stu.gxnu.edu.cn (Y.Y.); liuxh@stu.gxnu.edu.cn (X.L.); 13152591804@163.com (S.H.); denghua@mailbox.gxnu.edu.cn (H.D.); 2 Key Laboratory of Ecology of Rare and Endangered Species and Environmental Protection, College of Environment and Resources, Guangxi Normal University, Guilin 541004, China; 3 College of Civil Engineering and Architecture, Guilin University of Technology, Guilin 541004, China; 1020210576@glut.edu.cn (J.J.); 1020210588@glut.edu.cn (C.W.)

**Keywords:** plantation forest, wildfire, enzyme activity, carbon fraction, bacterial community

## Abstract

In order to study the effects of wildfires on soil carbon dioxide (CO_2_) emissions and microbial communities in planted forests, *Pinus massoniana* Lamb. and *Cunninghamia lanceolata* (Lamb.) Hook. forests were selected as the research subjects. Through a culture test with 60 days of indoor constant temperature, the soil physical and chemical properties, organic carbon mineralization, organic carbon components, enzyme activity, and microbial community structure changes of the two plantations after fire were analyzed. The results showed that wildfires significantly reduced soil CO_2_ emissions from the *Pinus massoniana* forests and *Cunninghamia lanceolata* forests by 270.67 mg·kg^−1^ and 470.40 mg·kg^−1^, respectively, with *Cunninghamia lanceolata* forests exhibiting the greatest reduction in soil CO_2_ emissions compared to unburned soils. Bioinformatics analysis revealed that the abundance of soil *Proteobacteria* in the *Pinus massoniana* and *Cunninghamia lanceolata* forests decreased by 6.00% and 4.55%, respectively, after wildfires. Additionally, redundancy analysis indicated a significant positive correlation between *Proteobacteria* and soil CO_2_ emissions, suggesting that the decrease in *Proteobacteria* may inhibit soil CO_2_ emissions. The *Cunninghamia lanceolata* forests exhibited a significant increase in soil available nutrients and inhibition of enzyme activities after the wildfire. Additionally, soil CO_2_ emissions decreased more, indicating a stronger adaptive capacity to environmental changes following the wildfire. In summary, wildfire in the *Cunninghamia lanceolata* forests led to the most pronounced reduction in soil CO_2_ emissions, thereby mitigating soil carbon emissions in the region.

## 1. Introduction

Forest soil is a significant component of the forest ecosystem, comprising 16–26% of the global soil carbon pool [1]. Wildfires play a crucial role in forest ecosystems, and their frequency has increased due to global warming [2]. In 2022, China experienced a total of 709 wildfires, affecting approximately 4689.5 hectares of forest land [3]. Guangxi, a vital forest region in southern China, contributes to an annual carbon emission of approximately 1.19 × 10^4^ tons due to wildfires [4]. Numerous studies have demonstrated the significant impact of wildfires on soil carbon emissions [5,6,7]. Therefore, it is crucial to explore how wildfires affect soil CO_2_ emissions.

China’s planted forests account for approximately one third of the world’s total planted forest area, with planted forests in southern China accounting for more than 65% of the country’s carbon sinks [8]. However, planted forests are vulnerable to anthropogenic disturbances, which, in turn, affects soil carbon emissions [9]. Standardizing the management of planted forests can effectively enhance the carbon sequestration potential of forest soils. *Pinus massoniana* and *Cunninghamia lanceolata* are the main indigenous tree species in southern China, and they are extensively planted in Guangxi [10]. Therefore, exploring the changes in soil properties and CO_2_ emissions from planted forests after wildfires is crucial for local ecological restoration and soil carbon sequestration potential.

Soil organic carbon (SOC) is a crucial factor influencing soil carbon emissions and carbon storage [11]. SOC is often divided into active and inert organic carbon, and active organic carbon is easily decomposed by microorganisms. The components of activated carbon typically include microbial biomass carbon (MBC), dissolved organic carbon (DOC), and readily oxidizable organic carbon (ROC), which serve as effective indicators of soil organic carbon mineralization dynamics [12]. Microbial community metabolism plays a crucial role in soil carbon mineralization and dynamics in soil nutrient dynamics [13]. Wildfires induce changes not only in soil structure and function but also in microbial community structure, which, in turn, affects soil CO_2_ emissions [6,14]. For instance, wildfires can directly alter the abundance and diversity of soil microorganisms, or indirectly modify the soil environment, thereby impacting microbial communities [15]. The high temperatures of wildfires lead to a decrease in soil microbial biomass [16]. In addition, Dooley et al. [7] pointed out that wildfires reduce soil microbial biomass at high temperatures, and a decrease in microbial community diversity may result in reduced soil CO_2_ emissions. Conversely, Zhou et al. [6] reported an increase in soil microbial diversity following wildfires, which, in turn, increased soil CO_2_ emissions. However, the impact of wildfires on soil CO_2_ emissions through the disturbance of microorganisms is still controversial. Therefore, analyzing changes in soil microbial community structure is crucial for understanding wildfire’s impact on soil CO_2_ emissions and post-disaster ecological restoration.

In this study, two representative local planted forest soils were selected as research subjects after wildfires and incubated at a room temperature of 25 °C for 60 days. The objectives of our study were as follows: (1) to analyze the changes in soil physicochemical properties, soil respiration, carbon fractions, and enzyme activities after the wildfire, and to explore the wildfire’s effects on soil organic carbon mineralization; and (2) to analyze the changes in soil microbial community structure and examine how these changes affect soil CO_2_ emissions.

## 2. Materials and Methods

### 2.1. Study Area and Soil Sampling

**Figure 1:**
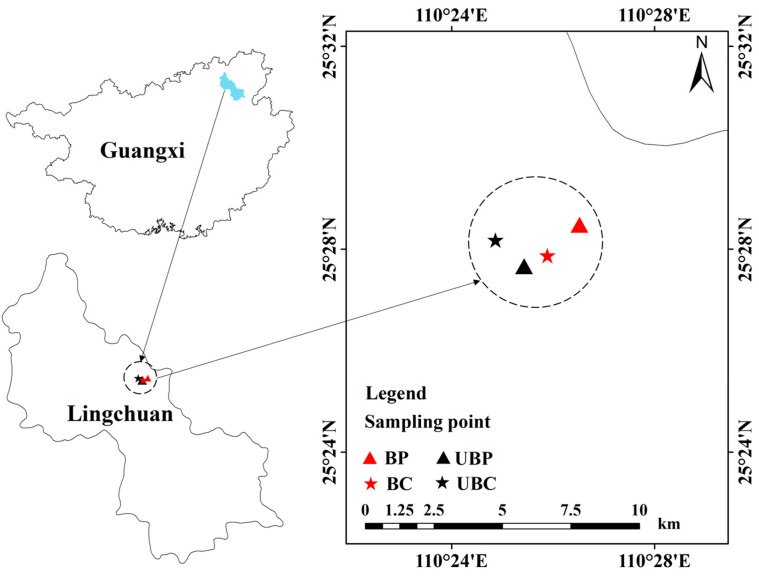
Study site and sampling design. Burned *pinus massoniana* (BP); unburned *pinus massoniana* (UBP); burned *cunninghamia lanceolata* (BC); unburned *cunninghamia lanceolata* (UBC).

The study site is located in Lingchuan County, Guilin City, Guangxi Zhuang Autonomous Region. The wildfire occurred on 17 October 2022, lasted for about four days, and burned approximately 16 hectares. The fire was caused by human activity and was of moderate intensity. The region has a subtropical monsoon climate, with an average annual rainfall of 1941.5 mm and an average annual temperature of 18.7 °C. According to the World Reference Base for Soil Resources standard, the soil type is classified as Fluvisols (IUSS Working Group WRB 2022) [17]. The sampling depth was 0–20 cm. The soils for our study were taken from two types of plantation forests typical of the area affected by the fire, namely, *Pinus massoniana* and *Cunninghamia lanceolata* forests. Details of the plantation forest types in the study area are shown in Table 1. The sampling sites are illustrated in Figure 1.

### 2.2. Soil Sampling

Sampling took place on 24 October 2022 and included burned *Pinus massoniana* forest (BP) and burned *Cunninghamia lanceolata* forest (BC), as well as adjacent unburned *Pinus massoniana* forest (UBP) and unburned *Cunninghamia lanceolata* forest (UBC). The unburned and burned plots had the same forest type and similar forest age, altitude, slope, and subtropical monsoon climate. The soil pH ranged from 4.71 to 5.31; the soil organic carbon content was similar. Therefore, it can be concluded that the samples collected from the unburned site can be equivalent to the soil samples collected from the fire site before the fire. In the unburned area, samples were collected after removing weeds and litter, while in the burned area, samples were taken after removing ash and thin litter layers. The sampling depth was 0–20 cm, and three sampling zones of 20 × 20 m were established for each forest type, with a total of 12 plots. The distance between plots was greater than 200 m to ensure the independence of data collection. Within each plot, five soil samples were collected and combined into a single sample. After removing the rhizomes and stones, the soil was allowed to dry naturally indoors, passed through a 2 mm nylon sieve, thoroughly mixed, and placed in self-sealing bags in a dry place protected from light for subsequent culture tests. Subsequently, the samples were used for incubation tests. Soil sampling was conducted during the incubation process, using sterile tools for high-throughput sequencing. The soil samples were divided into three sections. One portion was stored in a refrigerator at −4 °C for the determination of soil enzyme activity, dissolved organic carbon, and microbial biomass carbon. Another portion was stored in a sterile self-sealing bag at −80 °C, and then packaged in dry ice and sent to Sangon Biotech Co., Ltd., Shanghai, China, for high-throughput sequencing. The remaining soil was air-dried at room temperature, ground, and sieved through a 2 mm sieve for the determination of pH, available phosphorus, available potassium, alkali-hydrolyzed nitrogen, cation exchange capacity, organic carbon, and readily oxidizable organic carbon. The basic characteristics of the soil are shown in Table 2.

### 2.3. Experimental Design

This study was conducted in 2023 at the Key Laboratory of Ecology of Rare and Endangered Species and Environmental Protection (Guangxi Normal University), Ministry of Education, China. There were four treatments in this experiment, each replicated three times. First, 1 kg of air-dried soil sieved through a 2 mm mesh was placed in a 2 L polyethylene bottle, and deionized water was added to maintain 40% of the field capacity. To stimulate soil activity, the soil was pre-cultured at a temperature of 25 °C for 7 days. Following the pre-incubation period, soil samples were collected on days 1, 3, 5, 10, 15, 20, 30, 40, and 60 for subsequent analysis. Throughout this experimental period, water was replenished to maintain a field capacity of 40% using the weighing method. Additionally, another batch of soil samples (50 g each) was incubated under the same conditions for soil organic carbon mineralization analysis.

Soil organic carbon mineralization was measured as follows. First, 10 mL 0.5 mol·L^−1^ NaOH solution was placed in a small beaker inside a culture flask. Samples were taken on days 1, 3, 5, 10, 15, 20, 30, 40, and 60. The NaOH 5 mL was extracted and put into a triangular flask; 2 mL BaCl_2_ solution (1 mol·L^−1^) was added, and, then, 2 drops of phenolphthalein indicator were added. Finally, the solution was titrated with 0.25 mol·L^−1^ HCl until the red disappeared. The amount of CO_2_ absorbed by the NaOH solution was measured, and the amount of soil organic carbon mineralization was calculated based on the CO_2_ released by the soil. The absorption solution was replaced with a new one, and water was added to maintain 40% of the field capacity [18].

### 2.4. Measuring Indices

Soil pH was determined using deionized water extraction (soil-water ratio 1:2.5) and measured with a pH meter (FE28-Standard, Mettler Toledo, Shanghai, China) [19]. Available phosphorus (AP) was extracted with 0.03 mol·L^−1^NH_4_F-0.025 mol·L^−1^ HCl (1:7, *w*/*w*) and determined by UV-visible spectrophotometry (UV-1200, Mapada, Shanghai, China) [20]. Available potassium (AK) was extracted with 1 mol·L^−1^ NH_4_OAC (pH 7.0) (1:10, *w*/*w*) and determined by atomic absorption spectrophotometer (AA-6300, Analytik Jena, Jena, Germany) [21]. Alkaline nitrogen (AN) was determined using the alkaline diffusion method [22]. Cation exchange capacity (CEC) was measured using hexammine cobalt (III) chloride extraction-spectrophotometry [23]. Soil organic carbon (SOC) was determined using the determined by external heating method with potassium dichromate [20]. Dissolved organic carbon (DOC) was extracted with deionized water (soil-water ratio 1:5) and measured using a TOC analyzer (Multi N/C 3100, Analytik Jena, Jena, Germany) [20]. Microbial biomass carbon (MBC) was determined by fumigated with chloroform for 24 h, extracted with 1 mol·L^−1^ K_2_SO_4_ (1:4, *w*/*w*), and analyzed with TOC analyzer [24]. Readily oxidizable organic carbon (ROC) was determined by 333 mmol·L^−1^ potassium permanganate oxidation and sampling UV-visible spectrophotometer [25]. Catalase activity was determined by titration with potassium permanganate [26]. Soil urease activity was measured using urea as the substrate, incubating at 37 °C for 24 h, and determined by phenol-sodium hypochlorite coloration and UV-visible spectrophotometry [2]. Sucrase activity was measured using sucrose as the substrate, incubating at 37 °C for 24 h, and determined by the 3,5-dinitrosalicylic acid colorimetric method [26]. Soil CO_2_ emissions were measured using the alkali absorption method [18].

Soil DNA was extracted using the E.Z.N.A^TM^ Mag-Bind Soil DNA Kit (M5635-02, Omega, Norcross, GA, USA), following the manufacturer’s protocol. The quality of the extracted DNA was assessed by 1% agarose gel electrophoresis and UV spectrophotometry. The bacterial 16S rDNA V3-V4 region was amplified using primers 341F (5′-CCTACGGGGNGGCWGCAG-3′) and 805R (5′-GACTACHVGGGTATCTAATCC-3′) and sequenced on the Illumina MiSeq platform by Sangon Biotech Co., Ltd. (Shanghai, China) for high-throughput sequencing [27].

### 2.5. Calculations

Soil organic carbon mineralization (as CO_2_) can be obtained by the following equation [28]:

(1) $$CO_{2}\left(\mathrm{mg}\;\cdot \;kg^{-1}\right)=\left\{\left[\left(V_{0}-V\right)\times c\times 0.022\times \left(22.4/44\right)\times 1000\right]\times 2\times 1000\right\}/m$$

In Equation (1), V_0_ is the volume of standard hydrochloric acid consumed during blank titration, V is the volume of standard hydrochloric acid consumed during sample titration, c is the concentration of standard hydrochloric acid, 0.022 is the molar mass of carbon dioxide (1/2 CO_2_), M (1/2CO_2_) = 0.022 g·mmol^−1^, 22.4/44 is milliliters per gram of CO_2_ under standard conditions, and m is the weight of soil CO_2_ during incubation.

CO_2_ release rate (mg·kg^−1^·d^−1^) = amount of organic carbon mineralized/t, where t is the culture interval (d).

Cumulative mineralization of organic carbon can be obtained by the following equation:

(2) $$\mathrm{cumulative}\;\mathrm{mineralization}\;\mathrm{of}\;\mathrm{organic}\;\mathrm{carbon}=\sum_{1}^{n}CO_{2}$$

The soil carbon mineralization under different culture conditions was fitted by a first-order kinetic equation [28]:

(3) $$C_{t}=C_{0}\left(1\;-\;e^{-\mathrm{kt}}\right)$$

In Equation (3), C_t_ is the cumulative mineralization amount at culture time t (d), C_0_ is the potential soil carbon mineralization (mg·kg^−1^), k is the rate constant of soil carbon mineralization, d^−1^, and t is the culture time, d.

### 2.6. Statistical Analyses

Excel 2016 was used for data organization, and SPSS 22.0 was employed for one-way analysis of variance. The least significant difference (LSD) method was used to compare the differences between the data groups, and the statistical significance level was set as *p* < 0.05. Figures were plotted using Origin 2024. The valid data of the samples were clustered by OTU sequences using Uparse 7.0.1001 software at 97% similarity. Mothur 1.43.0 software was used to calculate the ACE index, Shannon index, and Simpson index of bacteria in the soil. Canoco 5.0 software was used to analyze the correlation between soil microbial colonies and environmental factors. R 4.3.3 corrplot package Pearson correlation analysis was used to analyze the direct correlation between soil chemical properties, carbon components, organic carbon mineralization, and enzyme activity. ArcGIS 10.2 was used to draw the sampling point map.

## 3. Results

### 3.1. Effect of Wildfires on Soil pH and Cation Exchange Capacity (CEC)

**Figure 2:**
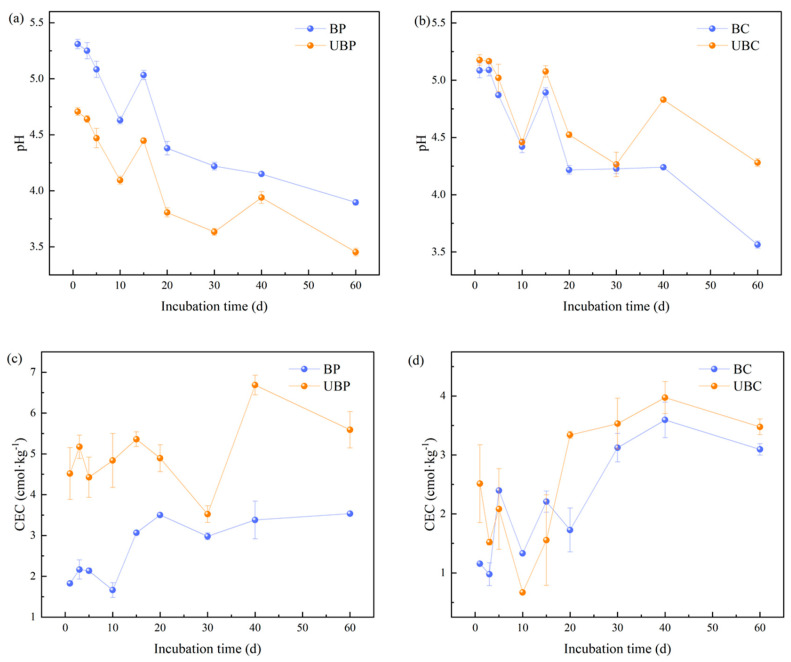
Effects of wildfire on soil pH and cation exchange capacity (CEC): (**a**,**c**) for *Pinus massoniana* forests; (**b**,**d**) for *Cunninghamia lanceolata* forests. Burned *pinus massoniana* (BP); unburned *pinus massoniana* (UBP); burned *cunninghamia lanceolata* (BC); unburned *cunninghamia lanceolata* (UBC).

The Soil pH of different standing soil types decreased with increasing incubation time, as shown in Figure 2a,b. Both burned and unburned soils in the same stands followed the same trend throughout the incubation period, with both showing an increase at day 15. At the end of the incubation, the soil pH of the *Cunninghamia lanceolata* stand decreased by 0.72 units. In contrast, the soil pH of the *Pinus massoniana* stand significantly increased, by 0.44 units.

Figure 2c,d shows that the CEC of the burned soil was reduced compared to the unburned soil. From days 20 to 60, the soil CEC of each stand demonstrated that the unburned soil had higher values than the burned soil, with all of them peaking at day 40. At the end of the incubation period, the burned *Cunninghamia lanceolata* forest soil CEC decreased the most, by 12.32%. The CEC of *Pinus massoniana* forest soil decreased by 58.11%. Overall, the wildfire reduced soil CEC.

### 3.2. Effects of Wildfires on Soil Nutrients

**Figure 3:**
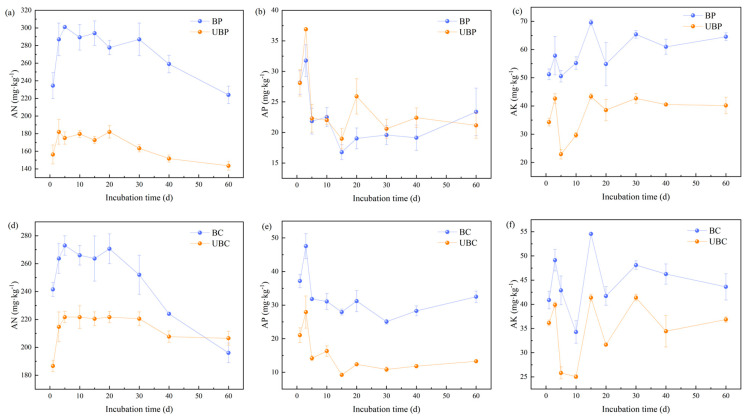
Effects of wildfires on soil nutrients: (**a**–**c**) for *Pinus massoniana* forests; (**d**–**f**) for *Cunninghamia lanceolata* forests. Burned *pinus massoniana* (BP); unburned *pinus massoniana* (UBP); burned *cunninghamia lanceolata* (BC); unburned *cunninghamia lanceolata* (UBC).

As shown in Figure 3a,b, wildfires significantly increased soil AN. Throughout the incubation period, the soil AN of both *Pinus massoniana* and *Cunninghamia lanceolata* forests exhibited an initial increase followed by a decline, peaking on the 5th day. At the end of the incubation, soil AN of burned *Pinus massoniana* forest increased by 56.10%. Conversely, burned *Cunninghamia lanceolata* soil, AN increased from day 0 to day 5 and then decreased rapidly after day 20.

The effects of wildfire on soil AP are shown in Figure 3c,d. In *Pinus massoniana* forest soil, the wildfire decreased soil AP. In contrast, compared to unburned *Cunninghamia lanceolata* forest soil, the wildfire significantly increased soil AP in the burned *Cunninghamia lanceolata* forest by 144.74% at the end of the incubation period.

Wildfires increase soil AK, as shown in Figure 3e,f. Both burned and unburned soils exhibited the same trend throughout the incubation period across different forest soil types. Soil AK peaked on day 15 in both the *Pinus massoniana* and *Cunninghamia lanceolata* forests. At the end of the incubation period, burning increased the AK in the soil of the *Pinus massoniana* forest by 60.67%, while in the *Cunninghamia lanceolata* forest, AK increased by 18.32%. Overall, wildfire raised soil AK.

### 3.3. Effects of Wildfires on Soil Organic Carbon Mineralization

**Figure 4:**
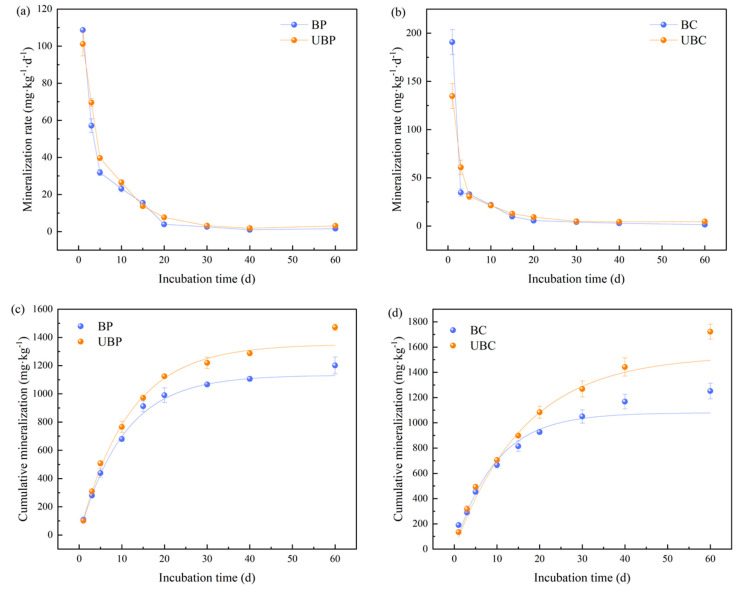
Effects of wildfire on soil organic carbon mineralization: (**a**,**c**) for *Pinus massoniana* forests; (**b**,**d**) for *Cunninghamia lanceolata* forests. Burned *pinus massoniana* (BP); unburned *pinus massoniana* (UBP); burned *cunninghamia lanceolata* (BC); unburned *cunninghamia lanceolata* (UBC).

As shown in Figure 4a,b, throughout the incubation period, the rate of organic carbon mineralization in all woodland soil types generally exhibited a rapid decline from 0–3 days, a slow decline from 3–20 days, and a gradual leveling off after 20 days. At day 1, the rate of mineralization was 1.94 and 3.33 times higher in unburned *Pinus massoniana* and *Cunninghamia lanceolata* than in burned *Pinus massoniana* and *Cunninghamia lanceolata* soils, respectively.

The effect of wildfire on the cumulative mineralization of soil organic carbon is shown in Figure 4c,d. Throughout the incubation period, the cumulative mineralization of organic carbon in all soil types gradually stabilized over time. During the period of 0–20 days, the cumulative mineralization increased more rapidly, while after 20 days, it gradually stabilized. At the end of the incubation period, the greatest decrease in the cumulative mineralization of soil organic carbon was observed in the burned *Cunninghamia lanceolata* forest, with a decrease of 37.59%, followed by that in the *Pinus massoniana* forest, with a decrease of 22.54%. In conclusion, wildfires could significantly reduce the cumulative mineralization of soil organic carbon.

The dynamics of cumulative organic carbon mineralization induced by wildfire across various soil types during the incubation period were analyzed using the first-order kinetic model C_t_ = C_0_(1 − e^−kt^). This model achieved a high fit, with a correlation coefficient of R^2^ ≥ 0.97 (Table S1). The results showed that the cumulative mineralization of organic carbon in different soil types followed the trend UBP > BP and UBC > BC. Conversely, the organic carbon turnover rate constant k exhibited the opposite pattern, with BP > UBP and BC > UBC.

### 3.4. Effects of Wildfires on Different Carbon Fractions of Soils

**Figure 5:**
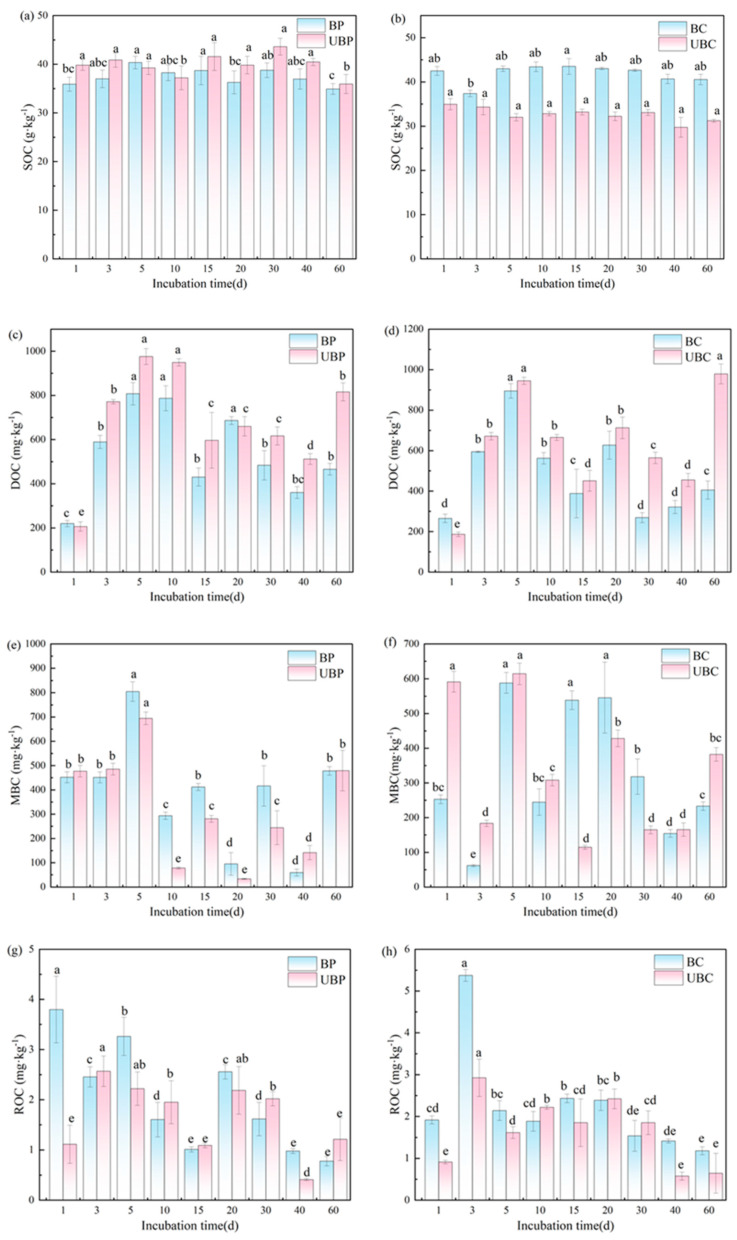
Effects of wildfires on SOC fractions: (**a**,**c**,**e**,**g**) for *Pinus massoniana* forest; (**b**,**d**,**f**,**h**) for *Cunninghamia lanceolata* forest. Burned *pinus massoniana* (BP); unburned *pinus massoniana* (UBP); burned *cunninghamia lanceolata* (BC); unburned *cunninghamia lanceolata* (UBC). Different lowercase letters indicate significant differences (*p* < 0.05).

Figure 5a,b shows that wildfires have different effects on SOC in different stands. In the *Cunninghamia lanceolata* forest, the burned SOC was greater than the unburned SOC. Meanwhile, the unburned SOC was greater than the burned SOC in the *Pinus massoniana* forest. At the end of the incubation, the SOC of the burned *Cunninghamia lanceolata* forest stands increased by up to 29.83%. In contrast, burning decreased the SOC of the *Pinus massoniana* forest by 2.85%.

As shown in Figure 5c,d, wildfires reduced soil DOC. Throughout the incubation period, the soil DOC of each stand soil type showed an increasing and then decreasing trend. On day 5, the soil DOC in the *Pinus massoniana* forest reached its peak. At the end of the incubation period, soil DOC decreased by 141.55% and 75.06% in the burned *Cunninghamia lanceolata* and *Pinus massoniana* forests, respectively.

Figure 5e,f demonstrates that the impact of wildfire on MBC varied across different plantation forests. In both the *Pinus massoniana* and *Cunninghamia lanceolata* forests, MBC exhibited an initial increase from days 0 to 5, followed by a gradual decline thereafter. Wildfires led to an increase in MBC in the *Pinus massoniana* forest soil. At the end of the incubation, the most significant decrease in MBC was observed in the soil of the burned *Cunninghamia lanceolata* forest, with a reduction of 63.97%. In contrast, MBC in the *Pinus massoniana* forest did not show significant changes.

Figure 5g,h shows a general trend of increasing and then decreasing soil ROC in different forests. In the *Cunninghamia lanceolata* forest, soil ROC peaked on day 3. In the *Pinus massoniana* forest, maximum ROC values were observed between days 1 and 3. At the end of the incubation, the soil ROC in the burned *Pinus massoniana* forest decreased by 56.03%. In contrast, burning significantly increased the ROC by 83.33% in the *Cunninghamia lanceolata* forest.

### 3.5. Effects of Wildfires on Soil Enzyme Activities

**Figure 6:**
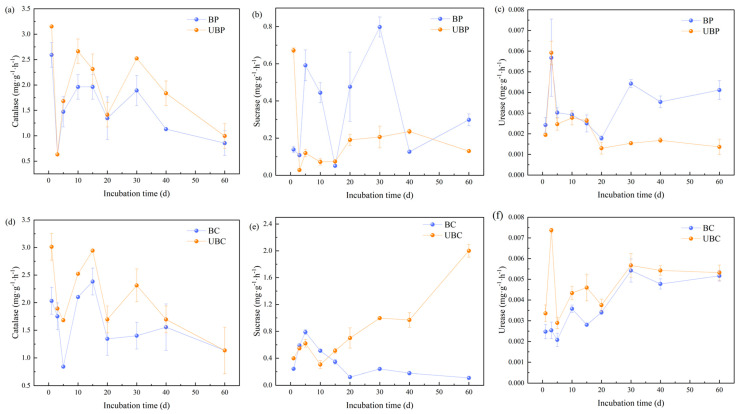
Effects of wildfire on soil enzyme activities: (**a**–**c**) for *Pinus massoniana* forests; (**d**–**f**) for *Cunninghamia lanceolata* forests. Burned *pinus massoniana* (BP); unburned *pinus massoniana* (UBP); burned *cunninghamia lanceolata* (BC); unburned *cunninghamia lanceolata* (UBC).

Figure 6a,b shows that wildfires reduced soil catalase activity. In all treatments, catalase activity decreased gradually with incubation time. At the end of incubation, the *Pinus massoniana* forest catalase decreased by 16.47%, while the unburned *Cunninghamia lanceolata* forest reached the same catalase activity as the burned *Cunninghamia lanceolata* forest.

The effects of wildfires on soil sucrase activity varied in different stands, as shown in Figure 6c,d. In the *Cunninghamia lanceolata* forest soil, soil sucrase activity increased 0–10 days after burning, while it decreased after 10 days. At the end of the incubation, burning reduced the soil sucrase activity in the *Cunninghamia lanceolata* forest soil by 18.18 folds. The overall trend of sucrase activity in the soil of the unburned *Pinus massoniana* forest showed a decreasing and then slowly increasing trend. In contrast, in the *Pinus massoniana* forest, burning showed a promoting effect on soil sucrase activity from 3 to 30 days.

The effect of wildfire on soil urease activity is shown in Figure 6e,f. At the end of the incubation, wildfire increased the soil urease activity of the *Pinus massoniana* forest by 66.99%. Conversely, in the burned *Cunninghamia lanceolata* forest, urease activity was suppressed, decreasing by 3.01% by the end of the incubation.

### 3.6. Effects of Wildfires on Bacterial Community Structure

The number of valid sequences obtained for all soil samples ranged from 37,328 to 42,837, and the number of bacterial OTUs ranged from a high of 1014 to a low of 1226, with a coverage of ≥0.995 (Table S2). The size of the Shannon index showed UBC > BP > UBP > BC. The Chao and ACE indices were indicated as UBC > BC > BP > UBP. Wildfire increased the diversity and richness of soil bacterial communities in the *Pinus massoniana* forest. Wildfire increased the diversity of bacterial communities in *Cunninghamia lanceolata* forest soils; however, it decreased soil bacterial community richness.

**Figure 7:**
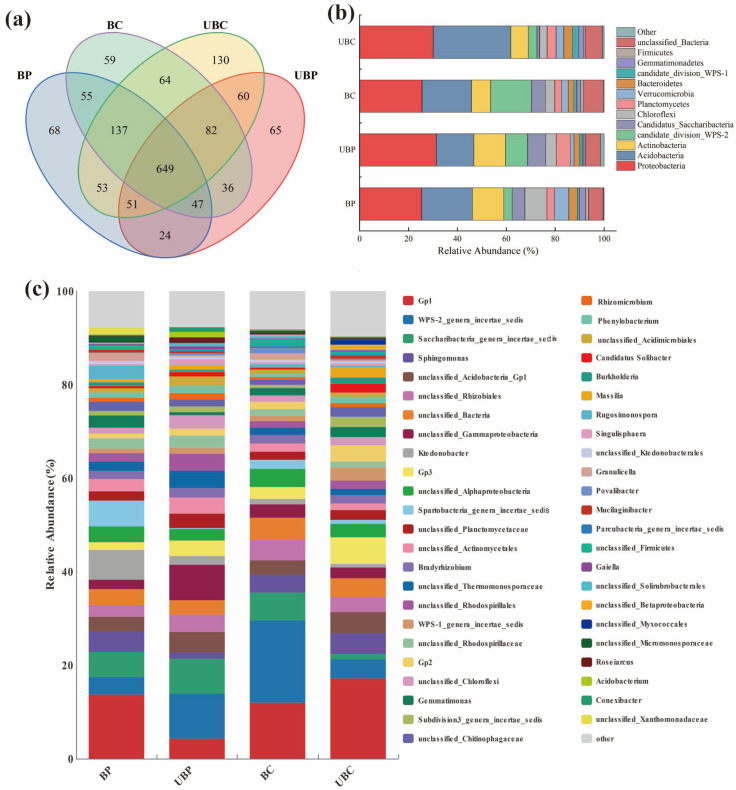
Effects of wildfires on soil microbial communities. (**a**) OUT Wayne plots of soil bacteria in different forest sites. (**b**) Relative abundance of soil bacteria at the phylum level in different forest sites. (**c**) Relative abundance of soil bacteria at the genus level in different forest stands. Burned *pinus massoniana* (BP); unburned *pinus massoniana* (UBP); burned *cunninghamia lanceolata* (BC); unburned *cunninghamia lanceolata* (UBC).

Figure 7a shows a total of 649 core OTUs in the two woodland soils, with the number of OTUs specific to each of the BP, UBP, BC, and UBC treatments being 68, 65, 59, and 130, respectively. The OTU sequences obtained were classified at the bacterial phylum level, and there were 13 major bacterial phyla (relative abundance > 0.01). Bacterial phyla with a relative abundance < 0.01 were merged into Others (Others). As shown in Figure 7b, *Proteobacteria* and *Acidobacteria* were the dominant bacteria in the four soils, accounting for 25.35–31.35% and 15.40–31.75%, respectively. Wildfire reduced the abundance of soil *Proteobacteria*, with the most significant decrease of 6.00% observed in *Pinus massoniana* forest soil. This was followed by a 4.60% decrease in *Cunninghamia lanceolata* forest soil. *Acidobacteria* and *Actinobacteria* accounted for the highest percentage of total bacterial phyla of the different forest soils, in addition to the *Proteobacteria*, amounting to 15.40–31.75% and 7.16–12.94%, respectively.

At the genus level, the following subgroups were obtained: *Acidobacteria Gp1* (3.36–15.03%), *WPS-2_genera_incertae_sedis* (3.43–16.73%), *Saccharibacteria_genera_incertae_sedis* (1.08–7.36%), *Sphingomonas* (1.21–1.06%), and *Spartobacteria_genera_incertae_sedis* (0.29–4.80%) (Figure 7c). Wildfire burned increased *Acidobacteria Gp1*, *Sphingomonas*, and Spartobacteria_genera_incertae_sedis by 8.93%, 2.81%, and 4.51%, respectively, in the *Pinus massonian* forest soil. In the *Cunninghamia lanceolata* forest soil, wildfire increased the abundance of *WPS-2_genera_incertae_sedis*, *Saccharibacteria_genera_incertae_sedis*, and *Spartobacteria_genera_incertae_sedis* by 13.21%, 4.64%, and 0.97%, respectively.

### 3.7. Correlation Analysis

**Figure 8:**
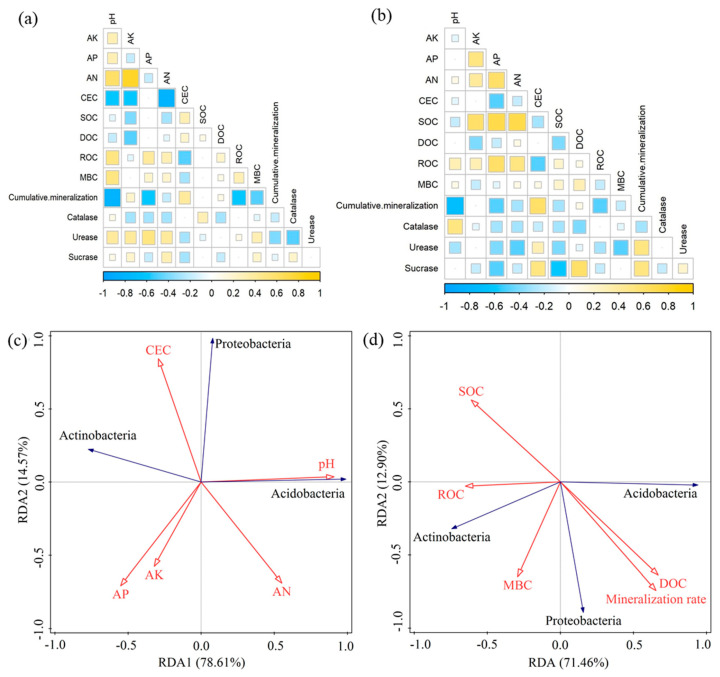
Correlation analysis. (**a**) *Pinus massoniana* forest; (**b**) *Cunninghamia lanceolata* forest; (**c**) effects of soil pH and quick nutrients on bacterial communities; and (**d**) effects of soil carbon fractions and mineralization rates on bacterial communities.

Figure 8a,b shows that in the *Pinus massoniana* forest soil, cumulative mineralization was significantly and negatively correlated with pH and ROC. In the *Cunninghamia lanceolata* forest soil, pH was significantly and negatively correlated with cumulative mineralization. Additionally, AN, AP, and AK were significantly and positively correlated with SOC, while ROC was significantly and negatively correlated with cumulative mineralization.

RDA analysis was employed to examine the interrelationships between soil chemical properties, carbon fractions, and mineralization rates in relation to the main dominant soil bacterial communities. Figure 8c shows that all soil chemical properties collectively accounted for 93.18% of the variance in soil bacterial community. Specifically, the first two axes of the RDA explained 78.61% and 14.57% of the total variance, respectively. CEC showed a significant positive correlation with *Proteobacteria*. *Acidobacterium* was significantly and positively correlated with soil pH. Figure 8d shows that the first two axes of the RDA explained 71.46% and 12.90% of the total variance, respectively, accounting for 84.36% of the total variation in bacterial community composition. *Proteobacteria* were also significantly and the positively correlated with DOC and organic carbon mineralization rate.

## 4. Discussion

### 4.1. Effects of Wildfires on Soil pH and CEC

Soil pH influences the availability of soil nutrients and is a critical indicator of soil quality [27]. Wildfires can modify the physical and chemical properties of topsoil through combustion heating and the deposition of aerodynamic ash [29]. Specifically, wildfires increase soil pH in *Pinus massoniana* forests. This can be explained by the fact that *Pinus massoniana* forests contain more pine needles, which are burned by wildfires and form a large amount of ash material on the surface, thus releasing more hydroxides as well as carbonates [30]. Wildfires decreased the soil pH in *Cunninghamia lanceolata* forests. This decrease may be attributed to the release of acidic humus from the decomposition of *Cunninghamia lanceolata* material after the fires [31]. In addition, root and microbial respiration in the forest floor leads to the dissolution of silicates, which results in a reduction in the alkaline cations, and, thus, in the soil pH [32]. Numerous studies have demonstrated that the decline in the soil CEC is related to the soil organic matter [33,34]. Wildfires lead to a reduction in soil organic matter, which can decrease soil CEC [35]. Significant declines in soil CEC were observed in both the *Pinus massoniana* and *Cunninghamia lanceolata* forests following wildfires. This reduction may also be attributed to thermal alterations in soil minerals caused by the high temperatures of the fires, which further contribute to decreased CEC [35].

### 4.2. Effects of Wildfires on Soil Nutrients

Soil available nutrients, which can be directly absorbed by plants, are a crucial indicator of soil nutrient availability [36]. Wildfire increased the AN levels in the *Cunninghamia lanceolata* forest soil, reaching a peak at the initial stage of incubation. This effect can be attributed to the formation of ash from burned litter, which then deposits into the soil and enhances nutrient levels [5]. Soil AN levels gradually decreased with the prolongation of incubation time. We hypothesize that increased microbial and enzyme activity over time accelerates nitrogen utilization. Qin et al. [37] observed, in their experimental study, that soil nitrogen gradually decreased over time, which is consistent with the findings of this study. Wildfire did not a significantly affect AP in the *Pinus massoniana* forest. This can be attributed to the strong adsorption capacity of the soil for phosphorus, which reduces the likelihood of phosphorus loss [38]. In contrast, in the *Cunninghamia lanceolata* forest soil, wildfire disturbance of the soil significantly increased AP. This effect may be due to root secretions from fir trees, which facilitate the conversion of less accessible phosphorus into a more readily available form [39]. Correlation results revealed a significant positive association between AP and soil organic carbon (SOC), indicating that AP increased in tandem with SOC. In our study, we observed that wildfires led to increased soil AK across all forested areas, with the most significant rise occurring in *Pinus massoniana* forests. This result contrasts with the findings of previous research [21]. We propose that the observed increase in AK may be attributed to enhanced degradation of soil organic matter under the high temperatures associated with wildfires [40]. On the other hand, wildfires can release potassium from both organic matter and apoplastic material in the soil in an inorganic form, leads to the deposition of potassium into the soil along with ash [41]. Additionally, wildfire significantly increased the available nutrients in the soil of the *Cunninghamia lanceolata* forest, indicating that the *Cunninghamia lanceolata* forest showed a stronger response to environmental changes after wildfire, which is beneficial for later ecological restoration.

### 4.3. Effects of Wildfires on Soil Organic Carbon Mineralization

Wildfires can alter the sources of carbon inputs, subsequently affecting the soil carbon cycle and carbon storage [42]. They significantly reduced soil CO_2_ emissions in all cases, with the greatest reduction observed in the *Cunninghamia lanceolata* forest soils. This reduction is attributed to wildfires reducing the amount of carbon available in the substrate, thereby decreasing CO_2_ emissions [43,44]. It is believed that this effect may be due to wildfires reducing the amount of carbon available in the substrate, thereby decreasing CO_2_ emissions. Additionally, methanogens may contribute to increased SOC fractions and enhanced respiration [45]. Wildfire significantly reduced the abundance of soil *Proteobacteria*, which may also have caused the decrease in soil CO_2_ emissions. Redundancy analysis showed that the rate of organic carbon mineralization was positively correlated with *Proteobacteria*, further suggesting that the decline in *Proteobacteria* may be responsible for the reduction in CO_2_ emissions. Additionally, the correlation results indicate a significant negative correlation between pH and cumulative mineralization, suggesting that changes in pH affect soil CO_2_ emissions. However, a study by Auwal et al. [46] reported that wildfires increase soil CO_2_ emissions, which is contrary to the results of this study. They suggest that this increase may be attributed to the elevated phosphorus limitation of the soil microbiota following wildfires, as well as the acceleration of microorganism metabolism due to the entry of apoplastic ash into the soil. The discrepancies between our findings and those of previous studies may be attributed to variations in tree species, climate [46], and soil types [47]. Our data are consistent with the views of [43,44]. In summary, the findings suggest that the carbon sequestration capacity of *Cunninghamia lanceolata* forests is enhanced after wildfires, indicating that planting *Cunninghamia lanceolata* can produce forests that can effectively respond to changes in the external environment and improve soil carbon sequestration potential.

### 4.4. Effects of Wildfires on Different Carbon Fractions of Soils

Wildfires significantly increased the SOC in *Cunninghamia lanceolata* forests while decreasing the SOC in *Pinus massoniana* forests. This effect may be attributed to the combined influence of apoplastic decomposition rate [48] and variations in bacterial abundance. In a study by Pérez-Cruzado et al. [49], disparities in SOC were observed between *Eucalyptus* and *Pinus massoniana* forests. These differences were attributed to variations in surface vegetation development between the two types of woodlands, which led to differential effects on SOC. The SOC of the *Cunninghamia lanceolata* forest increased, possibly due to the input of exogenous organic carbon released by fir tree apoptosis into the soil after the wildfire, thereby enhancing SOC [11,50]. The correlation results revealed a significant positive correlation between SOC and AK and AN in *Cunninghamia lanceolata* forests, suggesting that increases in AK and AN may lead to changes in SOC levels. In contrast, wildfire decreased SOC in the *Pinus massoniana* forest, possibly attributed to the high lipid content in pine needles, which are less readily decomposed by burning due to the species belonging to the coniferous forest, thus resulting in lower SOC [51]. Soil DOC can effectively reflect soil organic carbon mineralization [12]. The primary sources of soil DOC are surface litter and plant root secretions [52]. Wildfires significantly reduced soil DOC in both the *Cunninghamia lanceolata* and *Pinus massoniana* forests. This is consistent with the findings of [24], who suggested that this reduction may result from the decomposition of soil organic matter following wildfire disturbance, leading to a decrease in DOC. Yao et al. [52] suggested that wildfires reduce soil DOC by diminishing surface apoplastic material and killing plant roots. Additionally, it has been proposed that wildfires partially kill soil bacteria, thereby reducing microbial populations [53]. Furthermore, our high-throughput data indicated that wildfires resulted in a decrease in the abundance of *Proteobacteria* in the soil, further confirming this notion. Ma et al. [54] demonstrated a significant positive correlation between DOC and soil CO_2_ emissions. This suggests that the reduction in soil DOC due to wildfire consequently led to a decrease in soil CO_2_ emissions in both the *Pinus massoniana* and *Cunninghamia lanceolata* forests. Following the wildfire, the SOC in the *Cunninghamia lanceolata* forests increased, enhancing the carbon sequestration capacity of the soil.

In the *Pinus massoniana* and *Cunninghamia lanceolata* forests, soil MBC exhibited suppression during the pre-incubation period following wildfires, attributed to the inhibition of soil microorganism activity under the high-temperature conditions of the wildfire [15]. However, MBC showed an increase during the mid-culture period, consistent with findings reported by [55]. They attribute this to the decomposition of soil apoplastic material during the fire, which results in the ash containing unstable organic carbon that is gradually released into the soil over time. Wildfires promoted soil ROC in both woodland soils during the pre-cultivation period, possibly due to an increase in the amount of reactive organic matter resulting from the wildfires, enhancing soil organic carbon activity and leading to an increase in soil ROC [25]. Conversely, ROC gradually decreased in the later stages of incubation, likely due to increased soil microbial activity over time, leading to greater consumption of ROC [56].

### 4.5. Effects of Wildfires on Soil Enzyme Activities

Soil catalase, urease, and sucrase activities directly affect the soil carbon cycle and are key indicators of soil fertility [57]. Catalase catalyzes the decomposition of hydrogen peroxide in soil into water and oxygen, thereby reducing the damage to microorganisms [2]. Throughout the incubation period, wildfires reduced soil catalase activity across all forested areas. This effect can be attributed to two main factors: firstly, a decrease in soil microbial biomass following wildfire disturbance [58]; secondly, wildfires may reduce the substrate available to microorganisms, leading to a decrease in enzyme secretion by these microorganisms [59]. Additionally, the high temperatures associated with wildfires caused the degradation of many soil enzymes, which contributed to the decrease in catalase activity [60].

Soil sucrase catalyzes the decomposition of soil organic matter into glucose and sucrose, which can affect the turnover of soil carbohydrates [57]. Soil sucrase activity increased in the *Cunninghamia lanceolata* forest soil during the first 10 days of incubation following wildfire-induced burning. Burned branches and plants can serve as a nutrient source for soil microorganisms, thereby enhancing microbial activity and, subsequently, increasing sucrase activity [26]. However, soil sucrase activity was inhibited after 10 days following the wildfire. Wang et al. [61] have shown that sucrase activity decreases as soil pH decreases. This finding is further supported by our study, which observed that wildfire-induced burning reduced soil pH in the *Cunninghamia lanceolata* forest soil. In contrast, sucrase activity increased between 3 and 30 days in the burned *Pinus massoniana* forest soil. This increase may be due to elevated organic carbon levels and enhanced microbial metabolic activity in the burned soil having contributed to the higher sucrase activity [58].

Urease catalyzes the decomposition of soil urea into CO_2_ and ammonia [2]. During the pre-incubation period, wildfire inhibited soil urease activity in the *Pinus massoniana* forest. However, soil urease activity increased over time. We hypothesize that the microbial activity may have been improved over time, leading to increased urease activity. In contrast, wildfire inhibited soil urease activity in the *Cunninghamia lanceolata* forest. Wildfires destroy fine plant roots and root secretions, affecting below-ground carbon input and SOC uptake, which inhibits urease activity [47]. The correlation results indicated a negative correlation between urease activity and AN, suggesting that urease activity decreases as AN increases. Additionally, wildfires reduced the abundance of dominant bacterial phyla in the soil, inhibiting urease activity and resulting in decreased CO₂ emissions from the soil.

### 4.6. Effects of Wildfires on Soil Microbial Communities

Most soil microorganisms thrive in a pH range of 4–9; with the increase in pH, the microbial community diversity tends to increase, and pH and microbial community diversity are positively correlated [62]. Wildfires reduced microbial community diversity in the *Cunninghamia lanceolata* forest soil, likely due to a decrease in soil pH caused by the wildfire. In contrast, in the *Pinus massoniana* forest, wildfires increased microbial diversity, possibly due to an increase in soil pH. This aligns with other studies that report pH as a significant factor influencing microbial community diversity [63,64]. *Proteobacteria*, *Acidobacteria,* and *Actinobacteria* were the dominant bacterial phyla across all four soil types, consistent with the findings of previous studies [65,66]. Wildfires reduced the abundance of *Proteobacteria* in the soil, with the *Cunninghamia lanceolata* forest soil experiencing the smallest decrease. The decline in *Proteobacteria* abundance may be attributed to their high nutrient dependency. The burning of wildfires reduces nutrient accumulation in the soil, resulting in a nutrient-poor environment that, subsequently, diminishes *Proteobacteria* abundance [67]. Redundancy analysis revealed a positive correlation between CEC, DOC, and *Proteobacteria*, suggesting that wildfires decreased soil CEC and DOC, leading to a reduction *Proteobacteria* abundance. Similarly, wildfires were found to reduce *Aspergillus* abundance in the study [68]. The abundance of *Acidobacteria* in different soils consistently varied with soil pH, indicating a strong influence of soil pH on *Acidobacteria* abundance. Moreover, redundancy analysis showed a significant positive correlation between *Acidobacteria* and soil pH, further supporting this observation. These findings contrast with those of previous studies; some studies reported a negative correlation between *Acidobacteria* and soil pH [69], while others found no correlation [45]. Our study, further, observed that wildfires did not significantly affect soil *Acidobacteria* across various forest sites, a result consistent with the findings of [70]. It is generally believed that *Acidobacteria Gp1* is positively correlated with soil pH [71]. In this study, both forest soils were acidic, which may be the primary reason for the high abundance of *Acidobacteria Gp1*. Wildfire increased the abundance of soil *Sacchari-bacteria_genera_incertae_sedis* in the *Pinus massoniana* forest. This can be attributed to the fact that after the wildfire, additional carbon sources become available for the genus [72], thereby increasing its abundance. *Sphingomonas* is considered to play an important role in the soil carbon cycle [73]. In our study, *Sphingomonas* exhibited varying fluctuations in the two burned forest soils, which may contribute to the observed differences in CO_2_ emissions between the soils. In addition, *WPS-2_genera_incertae_sedis* and *Spartobacteria_ genera_ incertae_ sedis* are also core taxa in this study.

## 5. Conclusions

This study investigated the effects of wildfires on soil organic carbon sequestration and microbial communities in two typical plantation forest soils, both before and after wildfire events. Wildfires reduced soil CO_2_ emissions in both types of plantation forest soils, with the most significant reduction observed in the *Cunninghamia lanceolata* forest soil, which exhibited a stronger carbon sequestration capacity. Redundancy analysis revealed a significant positive correlation between soil CO_2_ emissions and *Proteobacteria*, suggesting that the decrease in *Proteobacteria* abundance following wildfires likely contributed to the suppression of soil CO_2_ emissions.

Wildfires increased the available phosphorus and potassium in the *Cunninghamia lanceolata* forest soil compared to the *Pinus massoniana* forest soil, indicating a stronger adaptive capacity to post-fire environmental changes. Furthermore, microbial biomass carbon and readily oxidizable organic carbon increased in the *Cunninghamia lanceolata* forest soil after the fire, while soil enzyme activities were inhibited. These changes contributed to enhanced carbon sequestration in the forest soils. These findings demonstrate that the *Cunninghamia lanceolata* forest soils are more adaptable to post-fire environmental changes and have a greater capacity for enhancing carbon sequestration. This provides a theoretical reference for soil carbon sequestration and ecological restoration in local plantation forests.

## Figures and Tables

**Table 1:** Basic information of the plantation forest types in the study area.

Plantation Types	BP	UBP	BC	UBC
Tree age (year)	16	15	15	16
Density (each plant·hm^−2^)	275	275	375	375
Latitude and longitude	25°30′33″ N 110°26′59″ E	25°29′59″ N 110°25′56″ E	25°30′01″ N 110°26′04″ E	25°30′24″ N 110°25′07″ E
Altitude (m)	254	221	233	212
Slope degree (°)	40	39	25	28
Slope position	Middle	Middle	Middle	Middle

Note: Burned pinus massoniana (BP); unburned pinus massoniana (UBP); burned cunninghamia lanceolata (BC); unburned cunninghamia lanceolata (UBC).

**Table 2:** Basic soil properties.

Sample	pH	SOC (g·kg^−1^)	AN (mg·kg^−1^)	AP (mg·kg^−1^)	AK (mg·kg^−1^)	CEC (cmol·kg^−1^)
BP	5.31 ± 0.04	35.89 ± 1.43	234.50 ± 14.85	28.16 ± 1.92	51.22 ± 1.87	1.83 ± 0.05
UBP	4.71 ± 0.04	39.82 ± 1.09	156.33 ± 10.69	28.10 ± 2.15	34.29 ± 1.37	4.52 ± 0.64
BC	5.09 ± 0.06	42.47 ± 0.99	241.50 ± 4.95	37.18 ± 1.98	40.88 ± 1.82	1.16 ± 0.01
UBC	5.18 ± 0.05	34.95 ± 1.27	186.67 ± 4.04	21.06 ± 2.17	36.16 ± 0.60	2.51 ± 0.12

Note: Burned *pinus massoniana* (BP); unburned *pinus massoniana* (UBP); burned *cunninghamia lanceolata* (BC); unburned *cunninghamia lanceolata* (UBC). AP, available phosphorus; AN, alkaline hydrolysis nitrogen; AK, available potassium; SOC, soil organic carbon; CEC, cation exchange capacity.

## Data Availability

Data are contained within the article.

## Supplementary Materials

The following supporting information can be downloaded at: https://www.mdpi.com/article/10.3390/microorganisms12081666/s1, Table S1. Kinetic parameters of soil organic carbon mineralization; Table S2. Analysis of soil microbial community structure.
