# Supplementary material for: Effects of Wildfire on Soil CO2 Emission and Bacterial Community in Plantations
Source: Microorganisms. 2024 Aug 13;12(8):1666. doi: 10.3390/microorganisms12081666 (PMC11357302; doi:10.3390/microorganisms12081666)
Supplement: Supplementary file 1 [file microorganisms-12-01666-s001.zip › microorganisms-3141979-supplementary.pdf]

Table S1. Kinetic parameters of soil organic carbon mineralization

Sample	Fitting Parameter		
	C_0/mg·kg^{-1}	k/d^{-1}	R^2
Burned *pinus massoniana* (BP)	1132.25±9.52*	0.096±0.003*	0.99
Unburned *pinus massoniana* (UBP)	1351.78±25.89*	0.086±0.005*	0.99
Burned *cunninghamia lanceolata* (BC)	1079.25±68.15	0.105±0.013*	0.97
Unburned *cunninghamia lanceolata* (UBC)	1533.74±165.36*	0.061± 0.011	0.98

Note: *p<0.05.

Table S2. Analysis of soil microbial community structure

Sample	Number of valid sequences	OTUs	Shannon	Simpson	Chao	Ace	Coverage
BP	41136	1084*	5.40*	0.010*	1262.33*	1256.56*	0.995
UBP	39974	1014	5.37*	0.012*	1153.58	1159.31	0.995
BC	42837	1129*	5.23	0.021*	1319.12*	1290.21*	0.995
UBC	37328	1226*	5.68*	0.009	1376.47*	1355.22*	0.995

Note: Burned *pinus massoniana* (BP); unburned *pinus massoniana* (UBP); burned *cunninghamia lanceolata* (BC); unburned *cunninghamia lanceolata* (UBC); *p<0.05.
